# Predicting potential microbe-disease associations with graph attention autoencoder, positive-unlabeled learning, and deep neural network

**DOI:** 10.3389/fmicb.2023.1244527

**Published:** 2023-09-18

**Authors:** Lihong Peng, Liangliang Huang, Geng Tian, Yan Wu, Guang Li, Jianying Cao, Peng Wang, Zejun Li, Lian Duan

**Affiliations:** ^1^School of Computer Science, Hunan University of Technology, Zhuzhou, China; ^2^College of Life Sciences and Chemistry, Hunan University of Technology, Zhuzhou, China; ^3^Geneis (Beijing) Co. Ltd., Beijing, China; ^4^Faculty of Pediatrics, The Chinese PLA General Hospital, Beijing, China; ^5^Department of Pediatric Surgery, The Seventh Medical Center of PLA General Hospital, Beijing, China; ^6^National Engineering Laboratory for Birth Defects Prevention and Control of Key Technology, Beijing, China; ^7^Beijing Key Laboratory of Pediatric Organ Failure, Beijing, China; ^8^School of Computer Science, Hunan Institute of Technology, Hengyang, China

**Keywords:** microbe-disease associations, graph attention autoencoder, positive-unlabeled learning, *K*-means, XGBoost, deep neural network

## Abstract

**Background:**

Microbes have dense linkages with human diseases. Balanced microorganisms protect human body against physiological disorders while unbalanced ones may cause diseases. Thus, identification of potential associations between microbes and diseases can contribute to the diagnosis and therapy of various complex diseases. Biological experiments for microbe–disease association (MDA) prediction are expensive, time-consuming, and labor-intensive.

**Methods:**

We developed a computational MDA prediction method called GPUDMDA by combining graph attention autoencoder, positive-unlabeled learning, and deep neural network. First, GPUDMDA computes disease similarity and microbe similarity matrices by integrating their functional similarity and Gaussian association profile kernel similarity, respectively. Next, it learns the feature representation of each microbe–disease pair using graph attention autoencoder based on the obtained disease similarity and microbe similarity matrices. Third, it selects a few reliable negative MDAs based on positive-unlabeled learning. Finally, it takes the learned MDA features and the selected negative MDAs as inputs and designed a deep neural network to predict potential MDAs.

**Results:**

GPUDMDA was compared with four state-of-the-art MDA identification models (i.e., MNNMDA, GATMDA, LRLSHMDA, and NTSHMDA) on the HMDAD and Disbiome databases under five-fold cross validations on microbes, diseases, and microbe-disease pairs. Under the three five-fold cross validations, GPUDMDA computed the best AUCs of 0.7121, 0.9454, and 0.9501 on the HMDAD database and 0.8372, 0.8908, and 0.8948 on the Disbiome database, respectively, outperforming the other four MDA prediction methods. Asthma is the most common chronic respiratory condition and affects ~339 million people worldwide. Inflammatory bowel disease is a class of globally chronic intestinal disease widely existed in the gut and gastrointestinal tract and extraintestinal organs of patients. Particularly, inflammatory bowel disease severely affects the growth and development of children. We used the proposed GPUDMDA method and found that *Enterobacter hormaechei* had potential associations with both asthma and inflammatory bowel disease and need further biological experimental validation.

**Conclusion:**

The proposed GPUDMDA demonstrated the powerful MDA prediction ability. We anticipate that GPUDMDA helps screen the therapeutic clues for microbe-related diseases.

## 1. Introduction

Microorganisms or microbes exist in the form of single cell or a group of cells. Microbes mainly contain bacteria, archaea, fungi, viruses, and protozoa (Wen et al., 2021). They widely distribute on the human skin, oral cavity, respiratory tract, and gastrointestinal tract (Holmes et al., 2015). Most of human microbes are beneficial to human health. They can promote nutrient absorption, protect human body against pathogens, and strengthen metabolic capability. In addition, they have the similar metabolic ability to the liver and are even taken as “forgotten organ” of human body (Gill et al., 2006). However, their imbalance or dysbiosis could cause human diseases (Peng et al., 2022c; Tian et al., 2022), such as inflammatory bowel disease (IBD) (El Mouzan et al., 2018), diabetes (Wen et al., 2008), asthma (Demirci et al., 2019), liver diseases (Henao-Mejia et al., 2013), and cancer (Schwabe and Jobin, 2013). Although many evidence demonstrated that microbes have close relationships with human diseases, a comprehensive understanding about how microbes influence human healths and produce diseases remains unknown.

Microbe–disease association (MDA) identification not only help us to capture the mechanisms of complex diseases but also provide multiple possible biomarkers for their diagnosis and therapy. However, traditional wet lab remains costly, laborious, and time-consuming (Chen et al., 2019, 2020; Shen et al., 2022; Chen and Huang, 2023). With the advance of single cell sequencing (Peng et al., 2022d, 2023a,b; Wu et al., 2022; Hu et al., 2023a,b; Xu et al., 2023) and wide application of artificial intelligence (Chen et al., 2021; Lihong et al., 2022; Peng et al., 2022a; Wang et al., 2022, 2023; Zhang et al., 2022a,b; Zhang and Wu, 2023), many computational methods have been developed to discover potential MDAs. These methods mainly contain network-based algorithms and machine learning-based algorithms.

Network-based algorithms take MDA prediction as a random walk or label propagation problem. For example, to decode underlying MDAs, BRWMDA fused similarity networks and bi-random walk (Yan et al., 2019), NBLPIHMDA developed a bidirectional label propagation algorithm (Wang et al., 2019), MHEN constructed a multiplex heterogeneous network (Ma and Jiang, 2020), WMGHMDA implemented iteratively weighted meta-graph search model (Long and Luo, 2019), RWHMDA was a hypergraph-based random walk method (Niu et al., 2019), BDHNS formulated a bi-directional heterogeneous MDA network (Guan et al., 2022), and MNNMDA used low-rank matrix completion (Liu et al., 2023).

Machine learning-based algorithms take MDA prediction as a classification problem. For example, to discover potential MDAs, BPNNHMDA (Li et al., 2020) adopted a neural network structure, GATMDA (Long et al., 2021) exploited a graph attention network with inductive matrix completion, DMFMDA (Liu et al., 2020) utilized a deep neural network-based deep matrix factorization model, NinimHMDA (Ma and Jiang, 2020) explored an end-to-end graph convolutional neural network structure, KGNMDA (Jiang et al., 2022) used a graph neural network model, MGATMDA (Liu et al., 2021) comprised decomposer, combiner, and predictor where the decomposer captured the latent components using node-level attention mechanism, the combiner obtained unified embedding using component-level attention mechanism, and unknown microbe–disease pairs were classified by a fully connected network. HNGFL (Wang et al., 2022) designed an embedding algorithm for feature learning and used support vector machine for MDA classification.

Although computational methods significantly improved MDA prediction and uncovered many potential MDAs, there are some limitations presented in this study. For example, network-based MDA inference methods cannot find associated entities for a new microbe or disease. Machine learning-based inference methods need reliable negative MDAs for implementing the MDA classification task. Here, we developed an MDA prediction method called GPUDMDA by combining feature extraction based on graph attention autoencoder (GATE), reliable negative MDA selection based on positive-unlabeled (PU) learning, and MDA classification based on deep neural network (DNN).

## 2. Materials and methods

### 2.1. Data preparation

We used two MDA databases to implement MDA prediction. One database is from the Human microbe–disease Association Database (HMDAD; http://www.cuilab.cn/hmdad) and contain 450 MDAs between 292 microbes and 39 diseases (Ma et al., 2017). The other comes from Disbiome (https://disbiome.ugent.be/home) (Janssens et al., 2018) and contains 4,351 MDAs between 218 diseases and 1,052 microbes. Moreover, an MDA network *Y*∈ℜ^*n*×*m*^ is constructed by Eq. (1) as follows:


(1) $$y_{ij}=\{\begin{array}{cc} 1, & \text{ if microbe }m_{i}\text{ associates with disease }d_{j}\\ 0, & \text{ otherwise } \end{array}$$

### 2.2. Pipeline for MDA prediction

**Figure 1:**
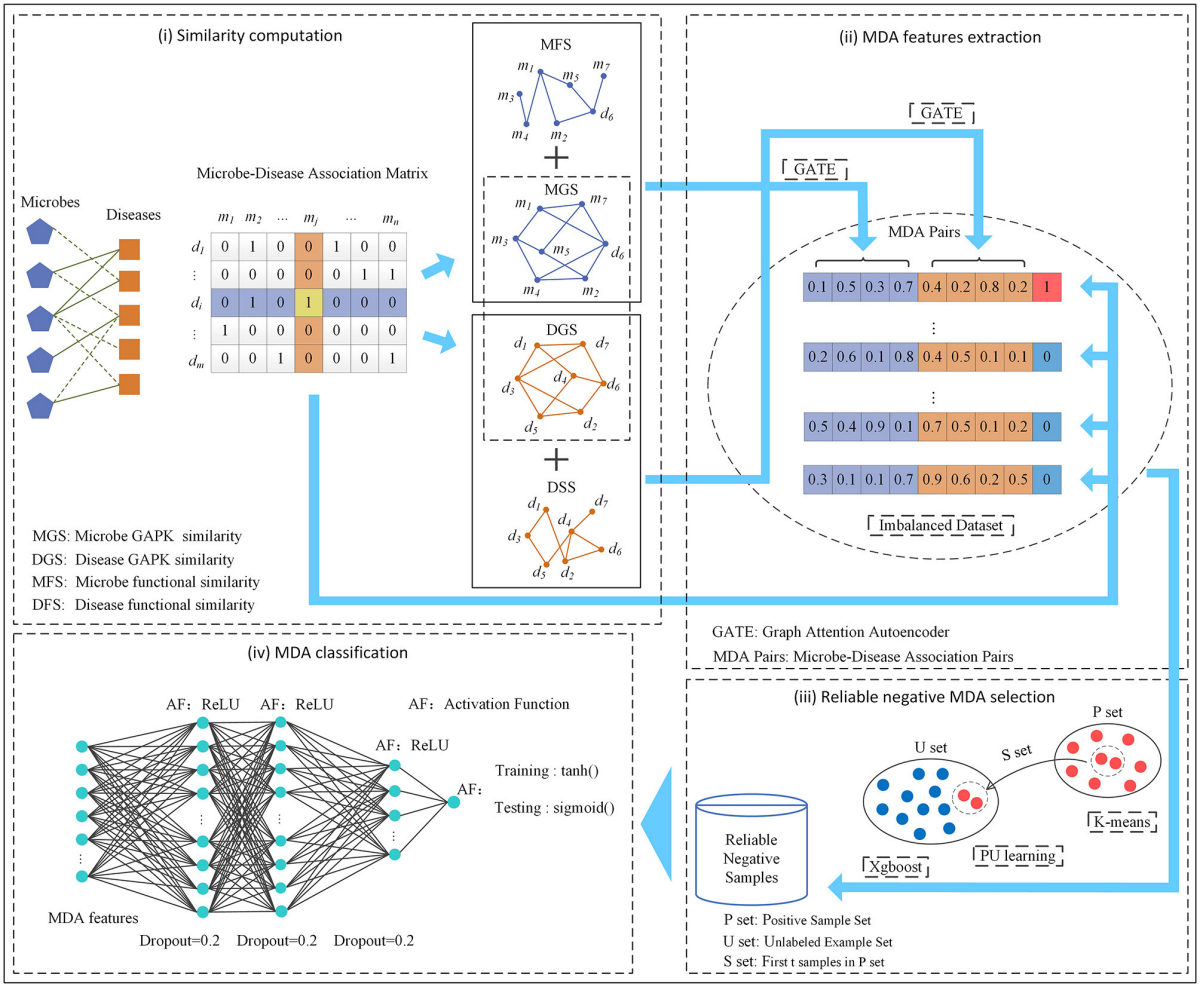
The pipeline of the MDA framework GPUDMDA.

In this manuscript, we developed an MDA prediction method called GPUDMDA by combining graph attention autoencoder, positive-unlabeled learning, and deep neural network. First, GPUDMDA computes disease similarity and microbe similarity matrices by integrating their functional similarity and Gaussian association profile kernel (GAPK) similarity, respectively. Next, it learns features of each microbe–disease pair using GATE. Third, it selects several reliable negative MDAs based on PU learning. Finally, it takes the extracted MDA features and the selected negative MDAs as inputs and proposes a DNN for discovering potential MDAs. Figure 1 shows the pipeline of GPUDMDA.

### 2.3. Similarity computation

#### 2.3.1. Functional similarity of microbes

In the GATMDA, Long et al. (2021) computed microbe functional similarity according to their co-occurrences (Kamneva, 2017). Similarly, we use the microbe function similarity method in GATMDA and then compute a functional similarity matrix $S_{m}^{fun}$ between *m* microbes, where $S_{m}^{fun}\left(m_{i},m_{j}\right)$ denotes the similarity between two microbes *m*_*i*_ and *m*_*j*_.

#### 2.3.2. Functional similarity of diseases

We use the disease functional similarity assessment method proposed by Long et al. (2021) and compute functional similarity matrix $S_{d}^{fun}$ between *n* diseases, where $S_{d}^{fun}\left(d_{i},d_{j}\right)$ denotes the similarity between two diseases *d*_*i*_ and *d*_*j*_.

#### 2.3.3. Gaussian association profile kernel similarity

GAPK function is a symmetric function along the radial direction. It can better cluster similar examples with linearly separable form (Wang et al., 2020). Let *V*_*d*_*i*__ (the *i*th row of *Y*) and *V*_*d*_*j*__ (the *j*th row of *Y*) denote two diseases *d*_*i*_ and *d*_*j*_, respectively, their similarity can be computed by Eq. (2) as follows:


(2) $$G_{d}(d_{i},d_{j})=\exp\left(-\theta_{d}{\left\|V_{d_{i}}-V_{d_{j}}\right\|}^{2}\right)$$

where


(3) $$\theta_{d}=\frac{1}{n}\sum_{i=1}^{n}\|V_{d_{i}}{\|}^{2}$$

Similarly, microbe GAPK similarity *G*_*m*_ is computed.

#### 2.3.4. Similarity integration

Functional similarity is used to measure microbe/disease similarity from the aspect of biological properties. GAPK similarity is used to evaluate microbe/disease similarity from the topological structure of MDA network. As compared with two individual similarity measurements, the combination of functional similarity and GAPK similarity can more accurately assess microbe/disease similarity and further improve MDA identification performance. Thus, we use the two types of information for microbe/disease similarity evaluation. Moreover, the final disease similarity matrix *S*_*d*_ is computed by integrating their functional similarity and GAPK similarity by Eq. (4) as follows:


(4) $$S_{d}\left(d_{i},d_{j}\right)=\left\{ \begin{array}{l} \frac{S_{d}^{fun}\left(d_{i},d_{j}\right)+G_{d}\left(d_{i},d_{j}\right)}{2}\text{ if }S_{d}^{fun}\left(d_{i},d_{j}\right)\neq 0\\ \;G_{d}\left(d_{i},d_{j}\right)\text{ otherwise. } \end{array}\right.$$

Similarly, microbe similarity matrix *S*_*m*_ is computed by Eq. (5) as follows:


(5) $$S_{m}\left(m_{i},m_{j}\right)=\left\{ \begin{array}{l} \frac{S_{m}^{fun}\left(m_{i},m_{j}\right)+G_{m}\left(m_{i},m_{j}\right)}{2}\text{ if }S_{m}^{fun}\left(m_{i},m_{j}\right)\neq 0\\ \;G_{m}\left(m_{i},m_{j}\right)\text{ otherwise. } \end{array}\right.$$

### 2.4. Feature extraction

GATE can efficiently learn features from structured graph data by stacking encoders and decoders (Deng et al., 2022). In this study, we use GATE to extract features for each microbe–disease pair. The GATE structure contain multiple encoders and decoders. In the encoders, each encoder uses a self-attention mechanism to generate new representations for nodes based on their neighborhood information (Veličković et al., 2017). In the *k*th layer of encoder, relationship between node *i* and its neighbor node *j* is computed by Eq. (6) as follows:


(6) $$\begin{array}{l} c_{ij}^{\left(k\right)}=\mathrm{Sigmoid}\left(V_{s}^{{\left(k\right)}^{T}}\sigma \left(W^{\left(k\right)}h_{i}^{\left(k-1\right)}\right)+V_{r}^{{\left(k\right)}^{T}}\sigma \left(W^{\left(k\right)}h_{j}^{\left(k-1\right)}\right)\right) \end{array}$$

where *W*^(*k*)^, $V_{s}^{\left(k\right)}$, and $V_{r}^{\left(k\right)}$ denote the trainable parameters in the *k*th layer of encoder with the sigmoid activation function. $h_{i}^{\left(k-1\right)}$ and $h_{j}^{\left(k-1\right)}$ denote the feature representations of nodes *i* and *j* in the (*k*−1)th layer, respectively. For the *i*th node, its associations with the other nodes are taken as its initial representation, that is, $h_{i}^{\left(0\right)}=x_{i}$, and its representation in the *k*th layer is generated by Eq. (7) as follows:


(7) $$\begin{array}{l} h_{i}^{\left(0\right)}=x_{i}h_{i}^{k}=\underset{j\in N_{i}}{\sum }\alpha_{ij}^{\left(k\right)}\sigma \left(W^{\left(k\right)}h_{j}^{\left(k-1\right)}\right) \end{array}$$

We use the softmax function to normalize coefficients of node *i*'s neighbors and solve the comparability problem by Eq. (8) as follows:


(8) $$\alpha_{ij}^{\left(k\right)}=\frac{\exp\left(c_{ij}^{\left(k\right)}\right)}{\sum_{l\in N_{i}}\exp\left(c_{il}^{\left(k\right)}\right)}$$

where *N*_*i*_ represents node *i* and its all neighbors. Moreover, the output in the final layer of encoder is considered the node representations.

In the decoder, the initial attributes of each node are reconstructed. Its input comes from the output in the final layer of encoder. Each neighbor of the current node is assigned to different weights by the attention mechanism. The normalized relevance between node *i* and its neighbor *j* in the *k*th layer of decoder is computed by Eqs (9) and (10) as follows:


(9) $${\hat{\alpha }}_{ij}^{\left(k\right)}=\frac{\exp\left({\hat{c}}_{ij}^{\left(k\right)}\right)}{\sum_{l\in N_{i}}\exp\left({\hat{c}}_{il}^{\left(k\right)}\right)}$$

(10) $${\hat{c}}_{ij}^{k}=\mathrm{Sigmoid}\left({\hat{V}}_{s}^{{\left(k\right)}^{T}}\sigma \left({\hat{W}}^{\left(k\right)}{\hat{h}}_{i}^{\left(k\right)}\right)+{\hat{V}}_{r}^{{\left(k\right)}^{T}}\sigma \left({\hat{W}}^{\left(k\right)}{\hat{h}}_{j}^{\left(k\right)}\right)\right)$$

where Ŵ^(*k*)^, ${\hat{V}}_{s}^{{\left(k\right)}^{T}}$, and ${\hat{V}}_{r}^{{\left(k\right)}^{T}}$ denote the trainable parameters in the *k*th layer of decoder. The *k*th layer in decoder reconstructs the node representations in the (*k*−1)th layer by Eq. (11) as follows:


(11) $${\hat{h}}_{i}^{k-1}=\sum_{j\in N_{i}}{\hat{\alpha }}_{ij}^{\left(k\right)}\sigma \left({\hat{W}}^{\left(k\right)}{\hat{h}}_{j}^{\left(k\right)}\right)$$

The loss function is defined by Eq. (12) as follows:


(12) $$Loss=\sum_{i=1}^{N}{\left\|x_{i}-{\hat{x}}_{i}\right\|}_{2}-\lambda \sum_{j\in N_{i}}\log\left(\frac{1}{1+\exp\left(-h_{i}^{T}h_{j}\right)}\right)$$

where the first and second terms denote the reconstruction loss of node features and one of graph structure, respectively. λ is a hyperparameter used to balance the contribution of two reconstruction loss terms. *x*_*i*_ and ${\hat{x}}_{i}$ represent the initial features and the reconstructed features of node *i*, respectively. *h*_*j*_ is the representation of a neighboring node *j* of node *i*.

Finally, we compute microbe feature vectors and disease feature vectors using GATE, and then, a microbe-disease pair is characterized as a *a*-dimensional vector by concatenating features of both the microbe and the disease.

### 2.5. Reliable negative MDA selection

In the area of machine learning, negative samples are equally important to final classification performance. However, there are lack of reliable negative MDAs on existing MDA databases due to the limitations of biological experiments. Thus, we design a reliable negative MDA selection method based on PU learning.

PU learning can efficiently identify high-quality negative samples from unlabeled samples and has been widely used in various practical situations (Li et al., 2022). The *K*-means clustering approach is one of the most popular unsupervised learning algorithms (Peng et al., 2022b). In the HMDAD and Disbiome databases, there are a few positive MDAs and multiple unknown microbe–disease pairs; that is, the two MDA databases are imbalanced. XGBoost has extremely fast parallel computation speed and demonstrates better performance in both balanced and imbalanced databases (Abdu-Aljabar and Awad, 2021).

In this manuscript, we propose a PU learning algorithm to select reliable negative MDAs by combining *K*-means clustering and XGBoost. Let that positive sample set *P* and unlabeled example set *U* denote known MDAs and unknown microbe–disease pairs, respectively. To select reliable negative MDAs from *U*, as shown in Algorithm 1, we design a PU learning algorithm.

**Algorithm 1:** A PU learning algorithm for selecting reliable negative MDAs.


1: Clustering each MDA sample with the *K*-means clustering algorithm based on the extracted MDA features using GATE.
2: Selecting the first *t* samples in *P* which have the smallest distance with cluster centroid as *S* and adding *S* into *U*.
2: Taking *P*−*S* as positive samples, and *U*+*S* as negative samples.
3: Calculating association score matrix *A* for all microbe–disease pairs based on XGBoost.
4: Ranking microbe–disease pairs in *S* based on association scores in *A* and obtaining the minimum score *A*_*min*_ in *S*.
5: For every sample *x* in *U*
6: If *A*_*x*_ satisfies *A*_*x*_<*A*_min_
7: then *RN* = *RN*∪*x*
8: Endfor
9: Obtaining reliable negative MDA samples *RN*.

Particularly, during PU learning, if spy samples are randomly selected from positive sample set *P* and placed into *U*, the obtained spy samples could be located at the boundary of the class cluster composed of samples in the entire *P* and belong to outliers. These spy samples have low spatial similarity with unknown positive examples in *U*. If a large number of noise or outliers are selected as spy samples, it will greatly affect the evaluation of the classifier on unlabeled samples, which could directly cause decreasing classification performance. Thus, we use *K*-means clustering algorithm for spy sample selection.

### 2.6. MDA prediction

We build a DNN to classify unknown microbe–disease pairs based on the extracted MDA features, the selected reliable negative MDAs, and known MDAs. The DNN contains an input layer, multiple hidden layers, and an output layer. In the input layer with *a* neurons, each MDA sample ***x*** with *a*-dimensional features is fed into the model by Eq. (13) as follows:


(13) $$\begin{array}{l} x=\left[x_{1},x_{2},\ldots ,x_{a}\right] \end{array}$$

where *x*_*i*_ denotes the *i*th feature in ***x***.

The *j*th hidden layer outputs the results by Eq. (14) as follows:


(14) $$\begin{array}{l} \begin{array}{c} \begin{array}{c} h_{j}=\overset{a}{\underset{i=1}{\sum }}w_{i}x_{i}+b_{j}\\ f\left(h_{j}\right)=ReLU\left(h_{j}\right) \end{array} \end{array} \end{array}$$

where *f* denotes the ReLU activation function. Finally, the output layer with the sigmoid activation function outputs MDA classification results by Eq. (15) as follows:


(15) $$\begin{array}{l} \sigma \left(h\right)=\frac{1}{1+e^{-h^{{}^{\prime }}}} \end{array}$$

where *h*′ denotes the output in the final hidden layer.

## 3. Result

### 3.1. Experimental settings

To evaluate the MDA prediction performance of our proposed GPUDMDA method, we compared it with other MDA identification methods (LRLSHMDA, NTSHMDA, GATMDA, and MNMDA) under five-fold cross validation (CV) on diseases, microbes, and microbe–disease pairs for 20 times. LRLSHMDA (Wang et al., 2017) is Laplacian regularized least square-based MDA identification algorithm, NTSHMDA (Luo and Long, 2018) is integrated random walk and network topology similarity, GATMDA (Long et al., 2021) combined inductive matrix completion and graph attention networks to complete missing MDAs, and MNNMDA (Liu et al., 2023) used a low-rank matrix completion model for identifying possible MDAs. During MDA prediction, it is not enough to reflect the MDA identification performance of a computational model only through cross-validation on microbe–disease pairs. Thus, in the study, we implemented cross-validations on microbes, diseases, and microbe–disease pairs to comprehensively assess the model's performance. The detailed definitions about the above three cross-validations have been proposed by Peng et al. (2020). AUC and AUPR were applied to measure the performance of MDA prediction methods.

In this study, we used GATE to extract features of microbes and diseases from their similarity networks, both of which are 64 dimensional vectors. We selected *t* samples from positive sample set *P* to place unlabeled example set *U*. When *t* was set to 15% of *P* on the HMDAD database and 20% of *P* on the Disbiome database, GPUDMDA obtained the best performance. Thus, we set *t* to 15 and 20% of *P* on the two databases, respectively. For DNN with four layers, the input layer, the following three hidden layer, and the output layer have 128, 100, 100, 50, and one nodes, respectively. Learning rate and “dropout” were set to 0.001 and 0.2. The parameter “epoch_num,” denoting the number of training, was set to 300 and 1,500 on the two databases, respectively. Disbiome is a larger dataset, and the proposed computational model needs to be trained for enough times to obtain better classification performance; thus, the “epoch_num” value was much larger on the Disbiome database.

Additionally, the number of positive samples is the same as one of the known MDAs.The number of selected credible negative MDAs is related to the computed smallest association probability score *A*_*min*_. Since the credible negative MDAs were selected from unknown microbe–disease pairs, unknown microbe–disease pairs were decreased but accounted for most of all microbe–disease pairs.

### 3.2. Performance comparison under CV on diseases

**Figure 2:**
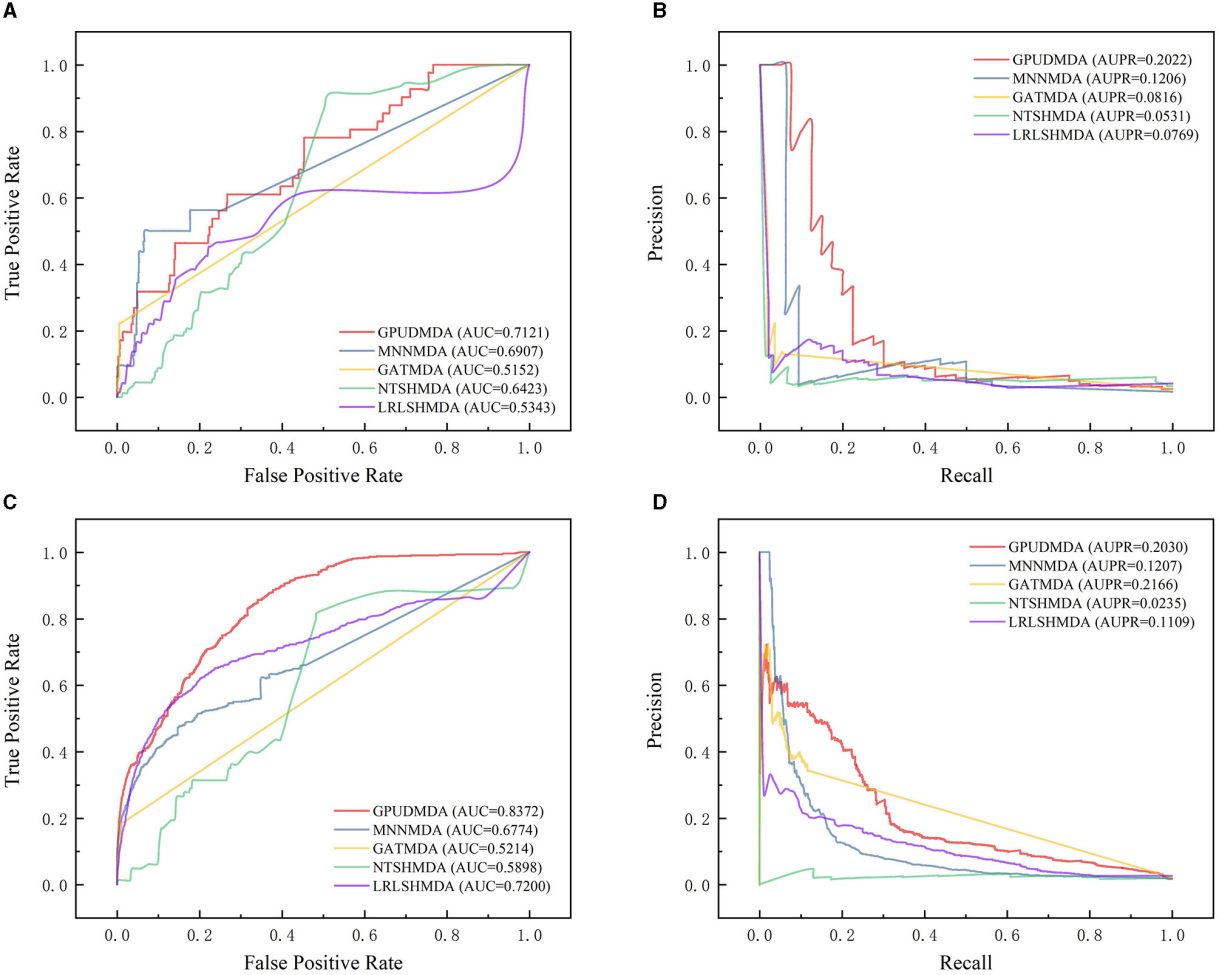
Performance comparison of five MDA prediction methods under five-fold CV on diseases. **(A, B)** The ROC and PR curves of the five methods on HMDAD. **(C, D)** The ROC and PR curves of the five methods on Disbiome.

Under CV on diseases, 80% diseases were taken as the training set and the remaining was test set. Figure 2 elucidates the receiver operating characteristic (ROC) and precision-recall (PR) curves of the five MDA prediction methods on the HMDAD and Disbiome databases under CV on diseases. Under CV on diseases, GPUDMDA obtained the best AUCs of 0.7121 and 0.8372, and the best AUPRs of 0.2022 and better AUPR of 0.2030 on the HMDAD and Disbiome databases, respectively, significantly outperforming LRLSHMDA, NTSHMDA, GATMDA, and MNMDA.

### 3.3. Performance comparison under CV on microbes

**Figure 3:**
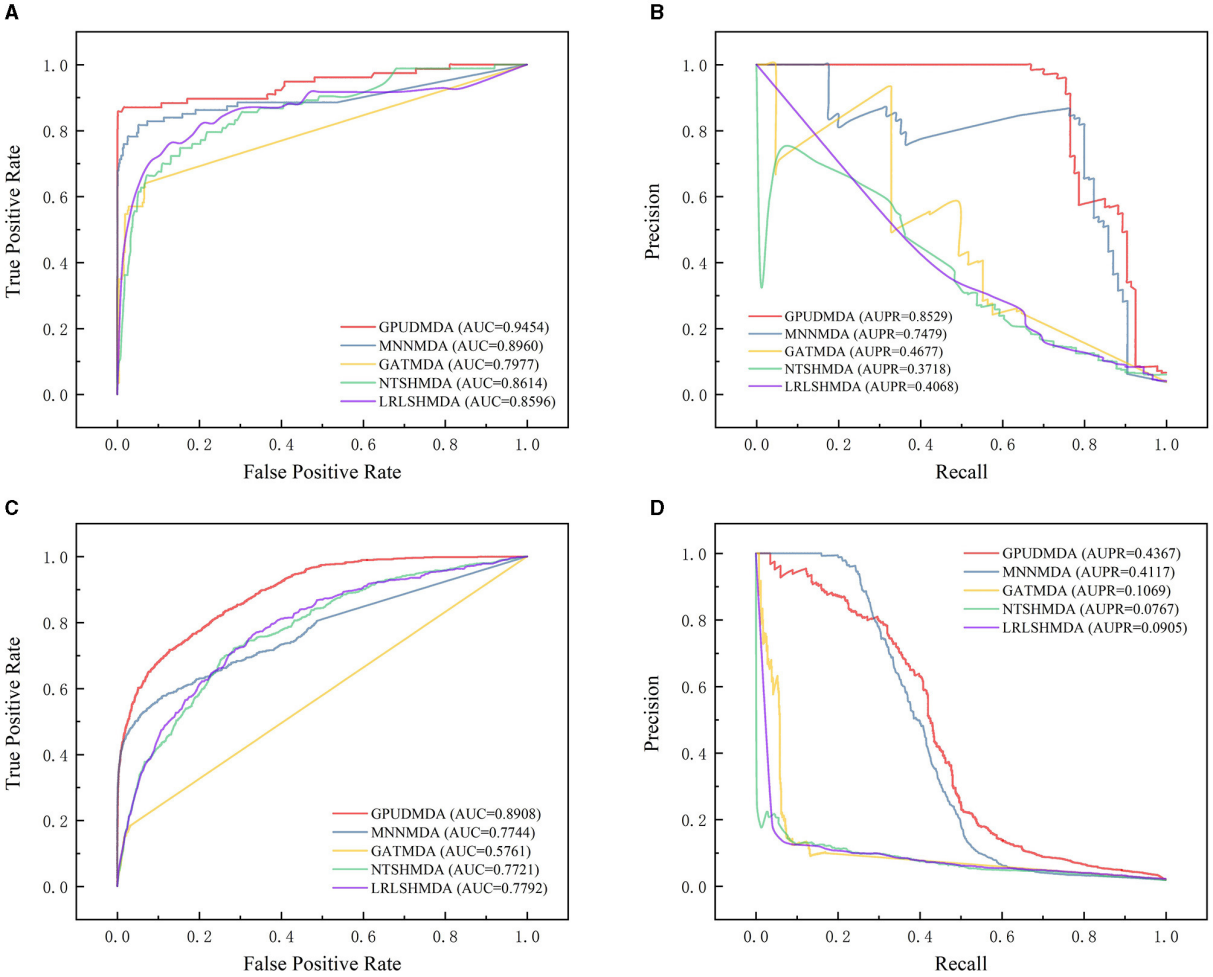
Performance comparison of five MDA prediction methods under five-fold CV on microbes. **(A, B)** The ROC and PR curves of the five methods on HMDAD. **(C, D)** The ROC and PR curves of the five methods on Disbiome.

Under CV on microbes, 80% microbes were taken as the training set and the remaining was test set. Figure 3 shows the ROC and PR curves of the five methods under CV on microbes. Under CV on microbes, GPUDMDA obtained better AUCs of 0.9454 and 0.8908 and AUPRs of 0.8529 and 0.4367 than LRLSHMDA, NTSHMDA, GATMDA, and MNMDA.

### 3.4. Performance comparison under CV on microbe–disease pairs

**Figure 4:**
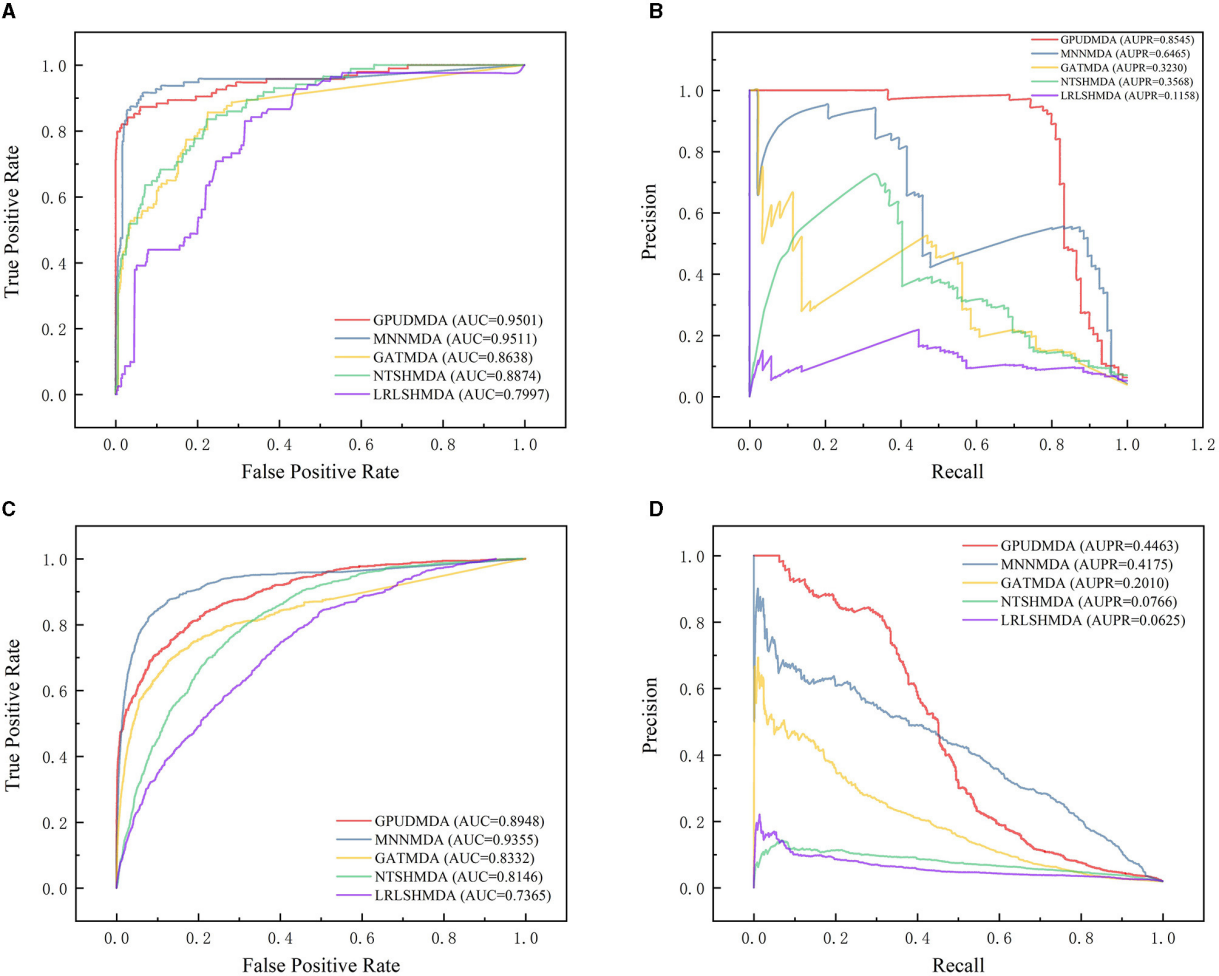
Performance comparison of the five MDA prediction methods under five-fold CV on microbe–disease pairs. **(A, B)** The ROC and PR curves of the five methods on HMDAD. **(C, D)** The ROC and PR curves of the five methods on Disbiome.

Under CV on microbe–disease pairs, 80% microbe–disease pairs were taken as the training set and the remaining was test set. Figure 4 illustrates the ROC and PR curves of the five MDA prediction methods under CV on microbe–disease pairs. Under the CV, GPUDMDA computed better AUCs of 0.9501 and 0.8948, and the best AUPRs of 0.8545 and 0.4464 among the five methods.

### 3.5. The affect of PU learning on performance

**Figure 5:**
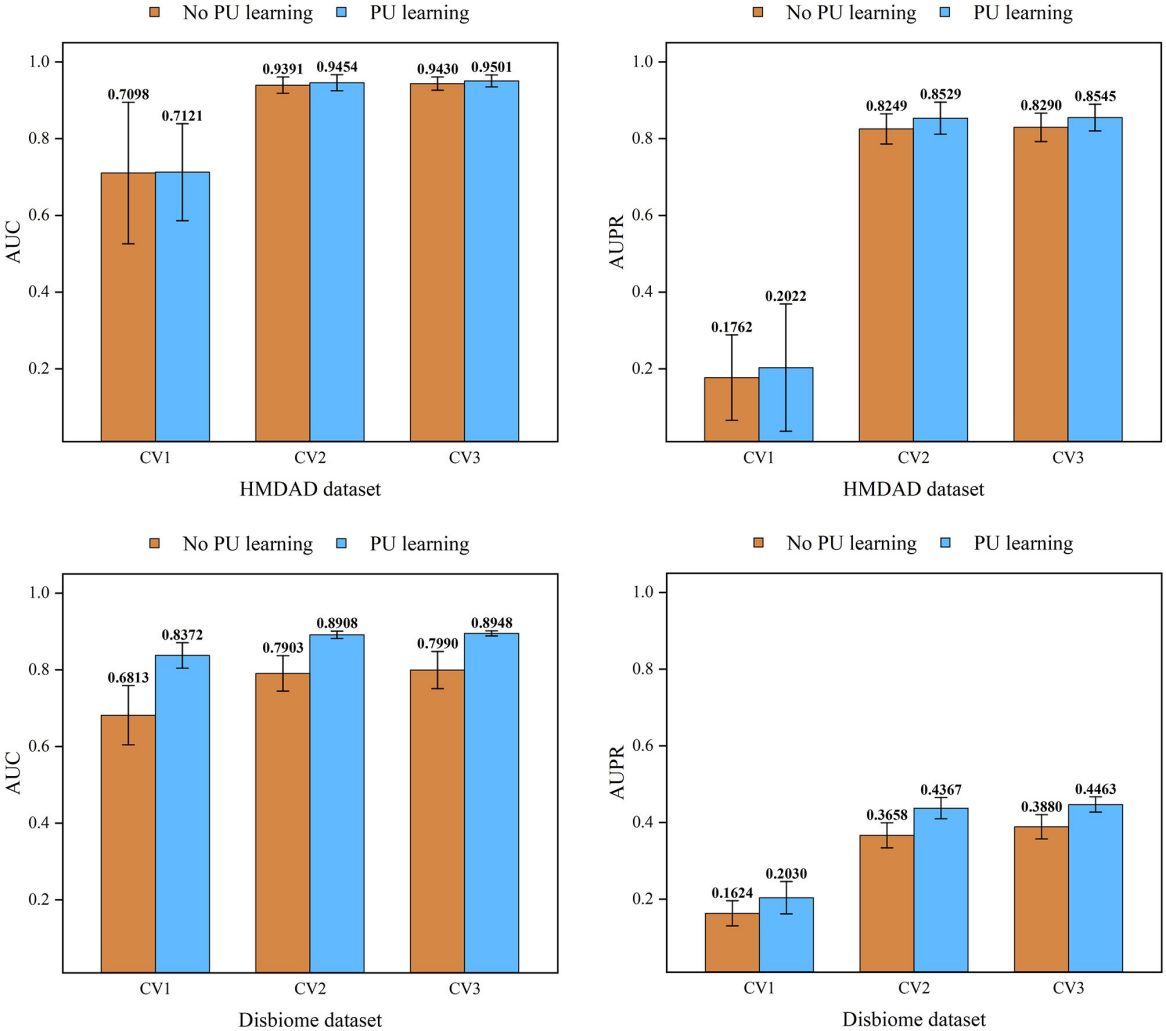
The impact of PU learning on performance in two databases.

Reliable negative samples can improve the classification performance of a model. To evaluate the reliability of the identified negative MDAs by GPUDMDA, we compared its performance under negative sample selection. Figure 5 demonstrates the affect of negative samples selected by PU learning on performance. The results elucidated that GPUDMDA with PU learning outperformed one without PU learning. Particularly, the performance of GPUDMDA with PU learning obtained significant improvement on Disbiome. The results suggested that reliable negative MDAs selected by PU learning can boost the MDA prediction ability.

### 3.6. Case study

In the above sections, we have confirmed the MDA identification accuracy of GPUDMDA. Next, we intend to find new microbes for asthma and IBD.

#### 3.6.1. Identifying new microbes for asthma

Asthma is a heterogeneous disease with respect to respiratory symptoms including chest tightness, shortness of breath, wheeze, and cough. It is the most common chronic respiratory condition and affects ~339 million people worldwide. Approximately 5%–10% of these patients have severe asthma. More than 10% of adults and 2.5% of children suffered from asthma have severe asthma (Brusselle and Koppelman, 2022; Reddel et al., 2022; Rattu et al., 2023).

We used the proposed GPUDMDA method to find new microbes associated with asthma. Tables 1, 2 show the predicted top 30 microbes that may associate with asthma on the HMDAD and Disbiome databases. The predicted 30 asthma-associated microbes included microbes with known association information with asthma and microbes without association information with asthma on the two databases. As shown in Table 1, 23 and 29 microbes can be validated by each or both of two databases or existing literatures among the identified top 30 potential asthma-associated microbes on the two databases, respectively. Furthermore, we found that *Enterobacter hormaechei* could associate with asthma with the ranking of 15 on the HMDAD database. On the Disbiome database, GPUDMDA predicted that *Enterobacter* may be a sole and unknown asthma-associated microbe among the predicted top 30 microbes associated with asthma.

**Table 1:** The predicted top 30 microbes associated with Asthma on HMDAD.

**Rank**	**Microbe**	**Evidence**
1	*Proteobacteria*	Confirmed by HMDAD and Disbiome
2	*Prevotella*	Confirmed by HMDAD and Disbiome
3	*Staphylococcus*	Confirmed by HMDAD and Disbiome
4	*Bacteroidetes*	Confirmed by HMDAD and Disbiome
5	*Enterobacteriaceae*	Confirmed by Disbiome
6	*Clostridium coccoides*	PMID: 1477358
7	*Firmicutes*	PMID: 23265859
8	*Comamonadaceae*	Confirmed by HMDAD and Disbiome
9	*Oxalobacteraceae*	Confirmed by HMDAD and Disbiome
10	*Sphingomonadaceae*	Confirmed by HMDAD and Disbiome
11	*Haemophilus*	Confirmed by HMDAD and Disbiome
12	*Helicobacter pylori*	Confirmed by HMDAD
13	*Enterococcus*	PMID: 29788027
14	*Enterobacter aerogenes*	PMID: 23842440
15	*Enterobacter hormaechei*	Unconfirmed
16	*Klebsiella pneumoniae*	PMID: 26953325
17	*Shigella dysenteriae*	Unconfirmed
18	*Lactobacillus*	Confirmed by Disbiome
19	*Clostridia*	PMID: 21477358
20	*Veillonella*	Confirmed by Disbiome
21	*Klebsiella*	Confirmed by Disbiome
22	*Prevotella copri*	Unconfirmed
23	*Actinobacteria*	PMID: 28947029
24	*Shuttleworthia*	Unconfirmed
25	*Desulfovibrio*	PMID: 29198875
26	*Clostridium difficile*	PMID: 21872915
27	*Oxalobacter formigenes*	Unconfirmed
28	*Fusobacteria*	Unconfirmed
29	*Porphyromonadaceae*	Confirmed by Disbiome
30	*Verrucomicrobiaceae*	Unconfirmed

**Table 2:** The predicted top 30 microbes associated with Asthma on Disbiome.

**Rank**	**Microbe**	**Evidence**
1	*Actinomyces*	Confirmed by Disbiome
2	*Bacteroides stercoris*	Confirmed by Disbiome
3	*Bifidobacterium*	Confirmed by Disbiome
4	*Blautia*	Confirmed by Disbiome
5	*Clostridiaceae*	Confirmed by Disbiome
6	*Clostridium neonatale*	Confirmed by Disbiome
7	*Comamonadaceae*	Confirmed by HMDAD and Disbiome
8	*Corynebacterium*	Confirmed by Disbiome
9	*Faecalibacterium*	Confirmed by Disbiome
10	*Gallibacterium*	Confirmed by Disbiome
11	*Gammaproteobacteria*	Confirmed by Disbiome
12	*Gemella*	Confirmed by Disbiome
13	*Klebsiella*	Confirmed by Disbiome
14	*Leclercia*	Confirmed by Disbiome
15	*Moraxella*	Confirmed by Disbiome
16	*Neisseria*	Confirmed by Disbiome
17	*Nitrosomonadaceae*	Confirmed by Disbiome
18	*Oxalobacteraceae*	Confirmed by HMDAD and Disbiome
19	*Planococcaceae*	Confirmed by Disbiome
20	*Prevotella*	Confirmed by HMDAD and Disbiome
21	*Pseudomonadaceae*	Confirmed by Disbiome
22	*Sphingomonadaceae*	Confirmed by HMDAD and Disbiome
23	*Staphylococcus*	Confirmed by HMDAD and Disbiome
24	*Stenotrophomonas*	Confirmed by Disbiome
25	*Streptococcus*	Confirmed by Disbiome
26	*Sutterella wadsworthensis*	Confirmed by Disbiome
27	*Veillonella*	Confirmed by Disbiome
28	*Weeksella*	Confirmed by Disbiome
29	*Propionibacterium*	PMID: 13268970
30	*Enterobacter*	Unconfirmed

*Enterobacter hormaechei* (Yeh et al., 2022) is a member and the most common nosocomial pathogen of the *Enterobacter cloacae* complex. It plays a key role in infectious diseases including, urinary tract infections, pneumonia, biliary tract infections, bacteremia, colitis and cellulitis. It is commonly found to be a high-pathogenicity island on its chromosome and is more virulent compared with other *E. cloacae* complex. In this study, GPUDMDA identified that *E. hormaechei* could associate with asthma.

**Figure 6:**
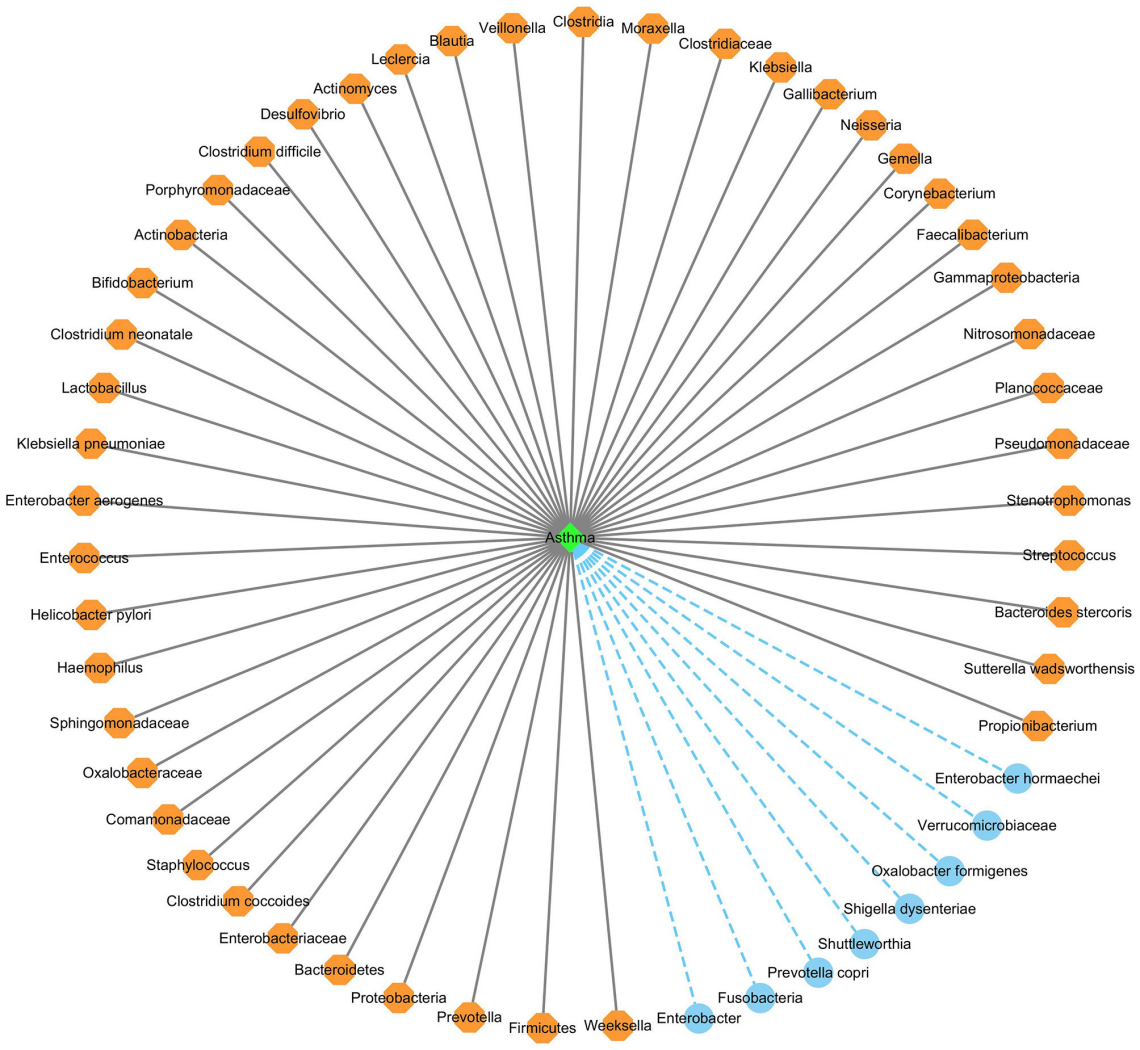
The predicted top 53 microbes associated with asthma on the two databases.

Figure 6 shows the association network between the predicted top 53 asthma-associated microbes and asthma, after removing the repeated associations on the two databases. In Figure 6, the gray solid lines and blue dashed lines denote known associations between microbes and asthma and the predicted associations between microbes and asthma, respectively.

#### 3.6.2. Identifying new microbes for inflammatory bowel disease

IBD is a class of globally chronic intestinal disease (Chang, 2020; Kaplan and Windsor, 2021). It widely exists in the gut and gastrointestinal tract and extraintestinal organs in many patients (Rogler et al., 2021). Up to 2 million Europeans and 1.5 million North Americans suffer from this disease (Jairath and Feagan, 2020). It mainly comprises Crohn's disease, ulcerative colitis, and indeterminate colitis (Flynn and Eisenstein, 2019). Many studies thought that it is the result of interactions between microbial, environmental, and immune-mediated factors. In particular, microbiome has been reported to have potential roles in the development, progression, and treatment of IBD. The gut microbiome is different in the IBD patients from one in healthy bodies (Glassner et al., 2020).

In particular, IBD is very common in children. Many pediatricians and the other pediatric clinicians meet children suffered from IBD. The IBD pediatric populations demonstrate the classic features of abdominal pain, bloody diarrhea, and weight loss as well as non-classic features of anemia, isolated poor growth, or the other extraintestinal symptoms. Recently, the IBD children patients show a rising incidence (Rosen et al., 2015; Oliveira and Monteiro, 2017). In total, 25%–30% of patients with Crohn's disease and 20% of patients with ulcerative colitis have been diagnosed in < 20 years of age. Moreover, 4% of pediatric IBD patients have been detected before 5 years (Kelsen and Baldassano, 2008). IBD severely affects normal growth and development of children. When treating children with newly diagnosed IBD, we need to consider their affects on growth and development and bone health (Rosen et al., 2015).

In this manuscript, we used the proposed GPUDMDA method to find potential microbes associated with IBD. Tables 3, 4 show the predicted top 30 IBD-associated microbes on the two MDA databases. The predicted 30 IBD-associated microbes included microbes with known association information with IBD and microbes without association information with IBD. In total, 20 and 28 predicted IBD-associated microbes can be validated by databases or existing publications among all predicted top 30 microbes on the two databases, respectively. On HMDAD, GPUDMDA predicted that *E. hormaechei* could associate with IBD with the ranking of 7. On Disbiome, the former 28 microbes have been confirmed to associate with IBD, and GPUDMDA also identified that *E. hormaechei* could link with IBD with the ranking of 29.

**Table 3:** The predicted top 30 microbes associated with IBD on HMDAD.

**Rank**	**Microbe**	**Evidence**
1	*Clostridium difficile*	PMID: 27499718
2	*Helicobacter pylori*	PMID: 22221289
3	*Staphylococcus*	PMID: 27239107
4	*Clostridia*	PMID: 31142855
5	*Clostridium coccoides*	PMID: 19235886
6	*Enterobacter aerogenes*	PMID: 4061480
7	*Enterobacter hormaechei*	Unconfirmed
8	*Klebsiella pneumoniae*	PMID: 9930068
9	*Shigella dysenteriae*	Unconfirmed
10	*Prevotella copri*	Unconfirmed
11	*Enterococcus*	PMID: 24629344
12	*Klebsiella*	PMID: 29573336
13	*Actinobacteria*	Confirmed by HMDAD
14	*Bifidobacterium*	PMID: 24478468
15	*Dietzia maris*	Unconfirmed
16	*Staphylococcus epidermidis*	Unconfirmed
17	*Oxalobacter formigenes*	Unconfirmed
18	*Tropheryma whipplei*	Unconfirmed
19	*Staphylococcus aureus*	PMID: 11424320
20	*Bacteroides vulgatus*	PMID: 29454108
21	*Actinomyces*	PMID: 30545401
22	*Porphyromonas gingivalis*	PMID: 31652577
23	*Selenomonas*	Unconfirmed
24	*Treponema*	PMID: 31851086
25	*Fusobacterium nucleatum*	PMID: 26718210
26	*Bacteroides ovatus*	PMID: 30666959
27	*Verrucomicrobiaceae*	PMID: 22572638
28	*Desulfovibrio*	Confirmed by Disbiome
29	*Clostridiales*	Unconfirmed
30	*Escherichia coli*	PMID: 29573336

**Table 4:** The predicted top 30 microbes associated with IBD on Disbiome.

**Rank**	**Microbe**	**Evidence**
1	*Anaerostipes*	Confirmed by Disbiome
2	*Bacillus licheniformis*	Confirmed by Disbiome
3	*Blautia*	Confirmed by Disbiome
4	*Bradyrhizobiaceae*	Confirmed by Disbiome
5	*Butyricimonas*	Confirmed by Disbiome
6	*Comamonadaceae*	Confirmed by Disbiome
7	*Christensenellaceae*	Confirmed by Disbiome
8	*Dehalobacter*	Confirmed by Disbiome
9	*Desulfovibrio*	Confirmed by Disbiome
10	*Dorea formicigenerans*	Confirmed by Disbiome
11	*Eubacterium biforme*	Confirmed by Disbiome
12	*Gemella*	Confirmed by Disbiome
13	*Gluconobacter oxydan*s	Confirmed by Disbiome
14	*Lachnobacterium*	Confirmed by Disbiome
15	*Methanobrevibacter smithii*	Confirmed by Disbiome
16	*Mogibacterium*	Confirmed by Disbiome
17	*Moraxellaceae*	Confirmed by Disbiome
18	*Pseudomonas straminea*	Confirmed by Disbiome
19	*Ruminococcus bromii*	Confirmed by Disbiome
20	*Saccharomyces cerevisiae*	Confirmed by Disbiome
21	*Streptococcus anginosus*	Confirmed by Disbiome
22	*Bacteroides ovatus*	PMID: 30666959
23	*Enterobacter aerogenes*	PMID: 4061480
24	*Fusobacterium*	PMID: 25307765
25	*Klebsiella pneumoniae*	PMID: 9930068
26	*Paraprevotella*	PMID: 25307765
27	*Propionibacterium acnes*	PMID: 28630242
28	*Staphylococcus*	PMID: 27239107
29	*Oxalobacteraceae*	Unconfirmed
30	*Enterobacter hormaechei*	Unconfirmed

**Figure 7:**
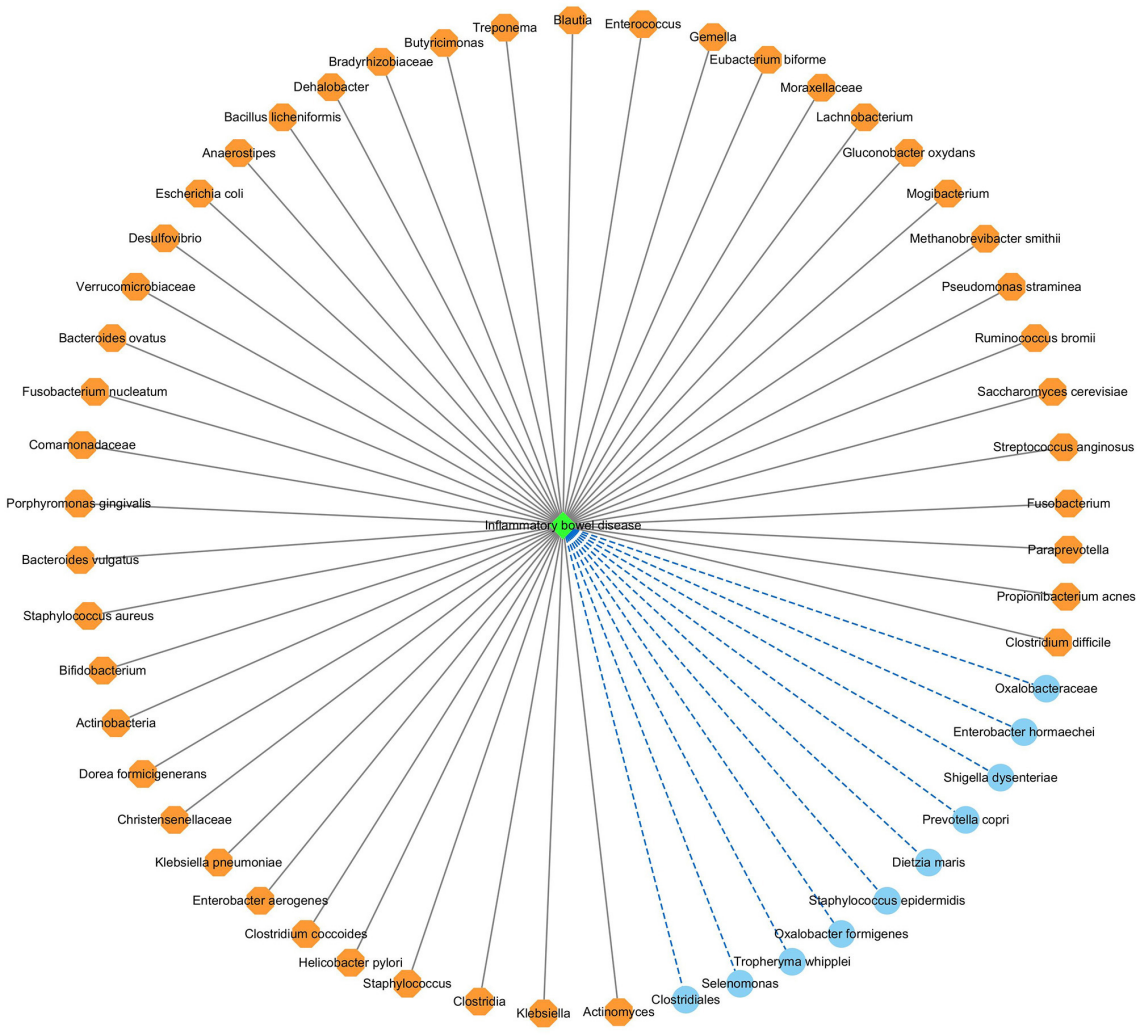
The predicted top 54 microbes associated with IBD on the two databases.

Figure 7 shows the association network between the predicted top 54 IBD-associated microbes and IBD, after removing the repeated associations on the two databases. In Figure 7, the gray solid lines and blue dashed lines denote known associations between microbes and IBD and the predicted associations between microbes and IBD, respectively.

## 4. Discussion and conclusion

Microbes manifest dense relationships with various human complex diseases. Predicting underlying MDAs can contribute to analyzing complex disease-causing mechanisms and screening potential biomarkers for the diagnosis and therapy of these diseases. Traditional wet lab methods are expensive, time-consuming, and laborious. Consequently, *in silico* methods have been increasingly developed as an efficient complementary to experimental methods.

In this study, we developed a deep learning model called GPUDMDA to capture new linkages between microbes and various human complex diseases. GPUDMDA first computed disease similarity and microbe similarity matrices based on their functional similarity and GIPK similarity, respectively. Next, it extracted features for each microbe–disease pair with GATE. Third, it selected a few reliable negative MDAs based on PU learning with *K*-means clustering and XGBoost. Finally, it took the extracted MDA features and the selected negative MDAs as inputs and designed a DNN to predict potential MDAs.

GPUDMDA was compared with four state-of-the-art MDA identification models (i.e., MNNMDA, GATMDA, LRLSHMDA, and NTSHMDA) on the HMDAD and Disbiome databases under five-fold CVs on microbes, diseases, and microbe–disease pairs. Under the three CVs, GPUDMDA computed the best AUCs and AUPRs on the two databases, suggesting that GPUDMDA could improve MDA prediction performance. Finally, we implemented case studies for asthma and IBD. The results showed that *E. hormaechei* could densely associate with asthma and IBD and need further biological experimental validation.

In future, we will combine biological features of microbe, diseases, and MDA network to design more accurate negative MDA selection method. In addition, we will also develop novel deep learning model to improve MDA classification performance based on the selected reliable negative MDA samples. Interestingly, we have conducted several computational models including existing classical MDA prediction methods. But the results elucidated that many models failed to compute better AUPR on the Disbiome database. It may be caused by different data structures of Disbiome. In future, we will further design a better robust computational method to improve MDA prediction on the Disbiome database. We hope that the proposed GPUDMDA method helps to identify microbes associated with related diseases and further contributes to mining the clues of treatment.

## Data availability statement

The original contributions presented in the study are included in the article/supplementary material, further inquiries can be directed to the corresponding authors. The HMDAD and Disbiome databases are available at: http://www.cuilab.cn/hmdad and https://disbiome.ugent.be/, respectively. Accession numbers can be downloaded at https://github.com/plhhnu/GPUDMDA.

## Author contributions

LP and LH: conceptualization and methodology. LP, ZL, and LD: funding acquisition. LP, GT, YW, ZL, and LD: project administration. LH: writing—original draft and software. LP, LH, PW, and ZL: writing—reviewing and editing. LP, LH, GT, GL, JC, and LD: investigation. LH, GL, JC, and LD: validation. All authors contributed to the article and approved the submitted version.
